# Reducing Dysfunctional Beliefs about Sleep Does Not Significantly Improve Insomnia in Cognitive Behavioral Therapy

**DOI:** 10.1371/journal.pone.0102565

**Published:** 2014-07-15

**Authors:** Isa Okajima, Shun Nakajima, Moeko Ochi, Yuichi Inoue

**Affiliations:** 1 Japan Somnology Center, Neuropsychiatric Research Institute, Tokyo, Japan; 2 Department of Somnology, Tokyo Medical University, Tokyo, Japan; 3 Yoyogi Sleep Disorder Center, Tokyo, Japan; University of Regensburg, Germany

## Abstract

The present study examined to examine whether improvement of insomnia is mediated by a reduction in sleep-related dysfunctional beliefs through cognitive behavioral therapy for insomnia. In total, 64 patients with chronic insomnia received cognitive behavioral therapy for insomnia consisting of 6 biweekly individual treatment sessions of 50 minutes in length. Participants were asked to complete the Athens Insomnia Scale and the Dysfunctional Beliefs and Attitudes about Sleep scale both at the baseline and at the end of treatment. The results showed that although cognitive behavioral therapy for insomnia greatly reduced individuals’ scores on both scales, the decrease in dysfunctional beliefs and attitudes about sleep with treatment did not seem to mediate improvement in insomnia. The findings suggest that sleep-related dysfunctional beliefs endorsed by patients with chronic insomnia may be attenuated by cognitive behavioral therapy for insomnia, but changes in such beliefs are not likely to play a crucial role in reducing the severity of insomnia.

## Introduction

Insomnia is defined as having difficulty initiating or maintaining sleep, waking up too early, or sleep that is chronically nonrestorative or poor in quality, causing daytime impairments such as decreased attention and worries concerning sleep [1]. Nearly 20% of the general adult population has been reported to suffer from insomnia [2], [3], and 10–15% of the individuals with insomnia show a chronic course [4], [5]. Chronic insomnia is associated with the development, treatment resistance to, and relapse of depression [4], [5], [6], [7].

Cognitive behavioral therapy for insomnia (CBT-I) is commonly used for treatment of the disorder. CBT-I has been shown to be effective for improving insomnia symptoms in 70–80% of patients with chronic insomnia [8], and to have long-term preventive effects on symptom recurrence [9]. Although a number of treatment outcome studies focusing on the CBT-I have been conducted [8], [10], [11], [12], only a few have investigated the underlying mechanisms or actual processes through which improvements after CBT-I occur [13], [14], [15]. Therefore, the specific reasons as to why CBT-I is effective for treating insomnia remain unclear.

Schwartz and Carney [15] reviewed theoretical models of insomnia and proposed several mediators of the treatment effectiveness of CBT-I. According to their hypothesis, behavioral (e.g., decreased time in bed), cognitive (e.g., decreased maladaptive beliefs about sleep), and hyperarousal (e.g., decreased physiological arousal) mediators are believed to account for therapeutic change via this intervention. There has been a surge of interest, in particular, in the role of sleep-related dysfunctional beliefs as potential perpetuating factor of insomnia. Some findings have suggested that such beliefs may be involved in exacerbation of the vicious cycle of insomnia [16]. Consequently, sleep-related dysfunctional beliefs have been incorporated into prominent cognitive behavioral models of insomnia [17].

Previous studies have examined the relationship between sleep-related dysfunctional beliefs and treatment outcomes with CBT-I [11], [13], [14], [18]. Based on these study results, Schwartz and Carney suggested that participants receiving CBT-I experienced (a) changes in their Dysfunctional Beliefs and Attitudes about Sleep (DBAS) from the baseline to the conclusion of treatment, as well as (b) greater reductions in DBAS scores as compared to the control group. They also proposed that (c) these reductions were associated with a range of subjective and objective sleep outcomes at the end of treatment and during the follow-up period [15]. This hypothesis implies that reductions in sleep-related dysfunctional beliefs are associated with positive changes in sleep outcomes with CBT-I. However, researchers have yet to examine if changes in sleep-related dysfunctional beliefs during CBT-I have a direct impact on improvement of insomnia. The cause-and-effect relationship between the reduction in DBAS and improvement of insomnia thus needs elucidating. Furthermore, although sleep efficiency and quality as determined from sleep diary data have been used as outcome variables in previous investigations [13], [14], [18], standardized measures of insomnia severity have not been examined in this regard. Therefore, the relationship between change in sleep-related dysfunctional beliefs and severity of insomnia has yet to be ascertained.

Considering these issues, the present study set out to clarify whether improvement of insomnia, as evaluated with the Athens Insomnia Scale (AIS), is mediated by a reduction in sleep-related dysfunctional beliefs through CBT-I.

## Materials and Methods

### Participants

Individuals were eligible for this study if they were 20 years old or above and met criteria for psychophisiological insomnia (i.e., chronic insomnia) according to the second edition of the International Classification of Sleep Disorders [1]. The participants of this study were outpatients who visited the Yoyogi Sleep Disorder Center seeking treatment for chronic insomnia between October 2011 and October 2012. Most of the patients were referred to the center by local psychiatrists or general practitioners. Participants’ disturbed sleep met all of the following additional criteria: (1) difficulties in initiating and/or maintaining sleep, defined as subjective sleep onset latency and/or waking after sleep onset greater than 30 minutes at least three nights per week, respectively [19]; (2) insomnia morbidity for over 6 months [19]; and (3) a score of 6 points or greater for severity of insomnia as measured with the AIS [20], [21]. Exclusion criteria were (1) insomnia due to medical or psychiatric disorders [22] or pharmaceutical use, and (2) the existence of other sleep disorders, such as obstructive sleep apnea syndrome, restless legs syndrome, periodic limb movement disorder, or circadian rhythm sleep disorders. In order to exclude these sleep disorders, after assessment by board-certified sleep disorder specialist physicians, eligible patients underwent nocturnal polysomnography (PSG) and/or provided self-checked sleep logs for more than 2 weeks, if necessary.

In all, 64 patients participated in the study; however, 11 patients answered at least one of the above-indicated scales incompletely. Therefore, the total number of participants with sufficient data for analysis was 53 (26 men, 27 women; mean [SD] age = 48.6 [15.1] years; mean [SD] duration of insomnia morbidity = 8.4 [11.4] years).

### Procedure

This study was approved by the ethical review board of the Neuropsychiatric Research Institute, Tokyo, Japan. Written informed consent was obtained from all participants. After diagnoses were made by board-certified sleep disorder specialist physicians, eligible participants were offered CBT-I [23]. Measures of insomnia severity and dysfunctional beliefs and attitudes about sleep were administered to patients during their first visit and immediately after treatment concluded.

### Measures

#### Athens Insomnia Scale (AIS) [20], [21], [24]

The AIS was used to assess the severity of insomnia according to criteria for insomnia outlined in the International Statistical Classification of Diseases and Related Health Problems, 10^th^ Revision (ICD-10) [25]. The AIS is an inventory consisting of eight items. The first five items assess nocturnal sleep problems (e.g., difficulty in sleep initiation, awakening during the night, early morning awakening), and the remaining three assess daytime dysfunction brought about by insomnia (e.g., overall functioning, sleepiness during the day). Responses are made on a 4-point scale ranging from 0 (*no problem at all*) to 3 (*very serious problem*). Participants were asked to rate corresponding items (e.g., awakenings during the night) as positive (i.e., to choose among rating options 1, 2, and 3) when they had experienced sleep difficulty at least three nights per week during the previous month. This scale was also used to measure concomitant change in insomnia symptoms over the course of treatment with CBT-I. A score of 6 points or greater on this scale indicates clinical levels of insomnia [20], [24].

#### Dysfunctional Beliefs and Attitudes about Sleep scale-16 (DBAS-16) [26], [27]

The DBAS has been utilized in previous mediation analyses, making it an effective tool for assessing the sleep-related dysfunctional beliefs of participants in our study. The DBAS-16 is a self-rating inventory consisting of 16 items (e.g., I am worried that I may lose control over my abilities to sleep) with 10-point scales, ranging from 0 (*strongly disagree*) to 10 (*strongly agree*). The total score is calculated from the average score of all the items on the scale and could range from 0 to 10, with higher scores indicating higher levels of dysfunctional beliefs about sleep.

### Treatment

The patients attended six biweekly individual treatment sessions, each lasting approximately 50 minutes. The first session began after the initial intake interview and case formulation. Core treatment components of CBT-I included sleep education and hygiene (Session 2), progressive muscle relaxation (Session 3), sleep scheduling (comprising sleep restriction and stimulus control, Sessions 4 and 5), and coping with worry (Session 6) based on the manual of a previous study [23]. Patients were asked to practice what they had learned and to track their results according to the treatment protocol between sessions.

### Data management and statistical analysis

Descriptive and inferential statistics were computed using SPSS version 19.0 (IBM Inc., Tokyo, Japan). The relationships between the scales at baseline and at the end of treatment were evaluated using paired *t*-tests and correlation analysis. The effect size (Cohen’s *d*) was used to investigate the extent of differences in scale scores between survey points (baseline vs. endpoint) in order to evaluate treatment effect. In general, Cohen's *d* values of 0.2 or lower, around 0.5, and 0.8 or more indicate small, moderate, and large effect sizes [28]. Effects of sleep-related dysfunctional beliefs on improvement of insomnia were assessed using path analyses following the steps outlined [29]. The severity of insomnia at baseline (AIS-T1) was adopted as the candidate predictor variable, change in sleep-related dysfunctional beliefs (i.e., difference in DBAS scores between baseline and endpoint) as the candidate mediator variable, and severity of insomnia after treatment (AIS-T2) as the criterion variable. We computed three regression analyses: First, we regressed AIS-T2 alone on AIS-T1; second, we regressed change in DBAS alone on AIS-T1; third, we regressed AIS-T2 on AIS-T1 simultaneously with change in DBAS. Typically, an effect size of 0.02 or below (coefficient of determination, *R^2^*) is considered small, whereas one around 0.13 is deemed moderate. One of 0.26 or greater is generally considered large [28]. Path analysis requires the usual assumptions of regression. Therefore, we assumed linear relationships among all variables and normally distributed error variables.

## Results

Means and standard deviations for clinical measures at baseline and at the end of the treatment are presented in Table 1. The results of paired *t*-tests showed significant decreases in participants’ AIS (*t*_52_ = 10.21, *p*<0.01) and DBAS (*t*_52_ = 3.69, *p*<0.01) scores at the end of treatment as compared to when they began. The effect sizes (*d*) were 1.21 and 0.72, respectively (see Table 1).

**Table 1:** Summary of self-reported measures of insomnia severity and sleep beliefs.

	Mean (SE)		Mean Difference score (*SE*)	*t* (*df*)	*p*	Effect size (95% CI)
	Baseline	End of treatment				
**DBAS total**	5.65 (0.18)	4.71 (0.25)	0.94 (0.25)	3.69 (52)	<0.01	0.72 (0.33, 1.11)
**AIS total**	11.96 (0.69)	5.89 (0.57)	6.08 (0.60)	10.21 (52)	<0.01	1.21 (0.80, 1.62)

*Note*. AIS = Athens Insomnia Scale; DBAS = Dysfunctional Beliefs and Attitudes about Sleep scale; SE = standard error; 95% CI = 95% Confidence Interval.

The results of correlation analyses showed significant correlations between: (1) the AIS and DBAS at baseline (*r* = 0.33, *p*<0.05), (2) AIS scores at baseline and at the end of treatment (*r* = 0.48, *p*<0.01), and (3) DBAS scores at baseline and at the end of treatment (*r* = 0.27, *p*<0.05) (refer to Table 2).

**Table 2:** Correlations between self-reported measures.

	DBAS_pre	AIS_post	DBAS_post
**AIS_pre**	0.33*	0.48**	0.19 n.s.
**DBAS_pre**	–	0.17 n.s.	0.27*
**AIS_post**		–	0.10 n.s.
**DBAS_post**			–

*Note*. AIS = Athens Insomnia Scale; DBAS = Dysfunctional Beliefs and Attitudes about Sleep scale; Pre = at baseline; Post = at the end of treatment;

**p<0.01,

*p<0.05.

**Figure 1:**
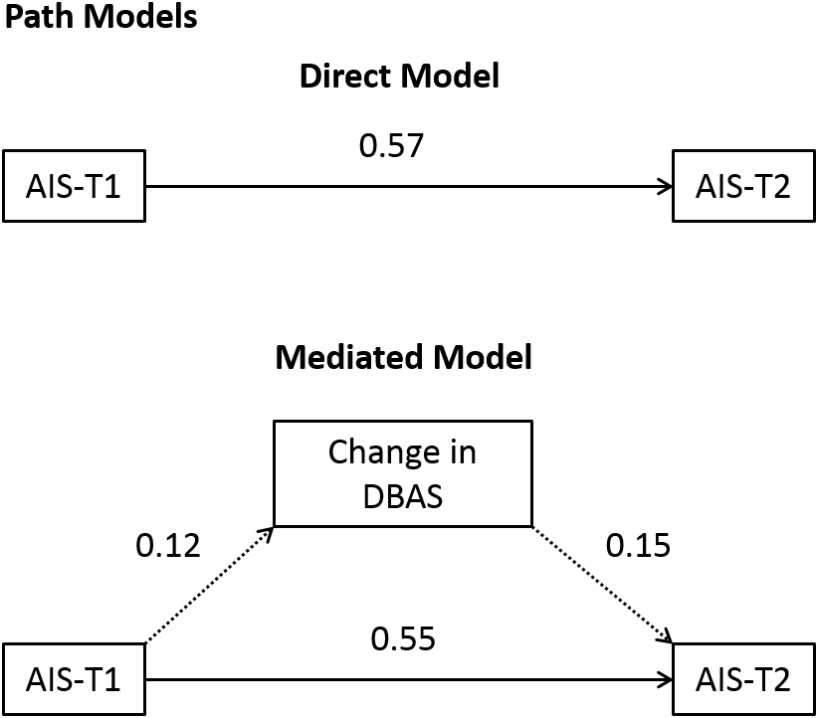
Two alternate path models. *Figure 1*. Two alternative path models, with severity of insomnia at baseline as the predictive variable of severity (of insomnia) at the end of treatment: a direct model, in which AIS-T1 has a direct effect on AIS-T2; and a mediated model, in which the effects of AIS-T1 on AIS-T2 are exerted via a change in dysfunctional sleep-related beliefs. Standardized regression coefficients (β) are listed for each path. The paths expressed with solid arrows are statistically significant (*p*<0.01), and those expressed with dashed arrows are not significant. AIS-T1 = Athens Insomnia Scale scores at baseline; AIS-T2 = Athens Insomnia Scale scores at the end of the treatment.

The first regression analysis revealed that AIS-T1 had a standardized direct effect on AIS-T2, with a *β*-value of 0.57 (*p* = 0.00; Model 1), but did not have a direct effect on change in DBAS (*p* = 0.41; Model 2). In the multiple regression equation with dysfunctional beliefs as the second independent variable (Model 3), the *β*-value of the AIS-T1 increased to 0.55 (*p* = 0.00), while change in DBAS did not show a direct effect on AIS-T2 (*β*-value = 0.15, *p* = 0.20; see Table 3). Path models using these variables are shown in Figure 1. Change in DBAS did not seem to mediate the effect of AIS-T1 on AIS-T2. The standardized path coefficient of 0.57 (Figure 1, Direct Model) decreased to 0.55 when change in DBAS was used as a mediator (Figure 1, Mediated Model). When change in DBAS was used as the second independent variable, the change in *R^2^* from 0.32 to 0.35 was significant (*ΔR^2^* = 0.03, *F*_2,50_ = 12.00, *p* = 0.00).

**Table 3:** Summary of path analyses.

*Regression for AIS-T2 with AIS-T1 (Model 1)*						
Model 1	β	*t*	*P*	*R^2^*	*ΔR^2^*	*p* (*ΔR^2^*)
**AIS-T1**	0.57	4.92	0.00	0.32	―	0.00
***Regression for dysfunctional beliefs*** a ***with AIS-T1 (Model 2)***						
**Model 2**	**β**	***t***	***p***	***R^2^***	***ΔR^2^***	***p*** ** (** ***ΔR^2^*** **)**
**AIS-T1**	0.12	0.82	0.41	0.01	―	0.41
***Multiple regression for AIS-T2 with AIS-T1 and dysfunctional beliefs (Model 3)***						
**Model 3** b	**β**	***t***	***p***	***R^2^***	***ΔR^2^*** ** (M3–M1)**	***p*** ** (** ***ΔR^2^*** **)**
**AIS-T1**	0.55	4.77	0.00	0.35	0.03	0.00
**Dysfunctional beliefs**	0.15	1.31	0.20			

*Note*. AIS = Athens Insomnia Scale; M = Model; *p* (*ΔR^2^*) = significance of change;

a indicates difference between DBAS scores at baseline and at the end of treatment;

b analysis of variance for Model 3 showed that multiple *R* was 0.57, multiple *R^2^* (adj.) was 0.30, and *F* ratio was 12.00 (*df_1_* = 2, *df_2_* = 50, *p* = 0.00).

## Discussion

The present study examined whether improvement in insomnia is mediated by a reduction in sleep-related dysfunctional beliefs as manifested by a decrease in DBAS score after CBT-I. The results suggest that DBAS scores decreased after CBT-I, but the change in these beliefs did not seem to play a crucial role in improving insomnia.

We compared the DBAS scores of insomnia patients in this study with those of good sleepers (*n* = 335, mean score = 2.96, *SD* = 0.13) and individuals with insomnia (*n* = 1049, mean score = 5.23, *SD* = 1.60) in a previous study involving a large sample [30]. We found that the scores of insomnia patients in the present study were significantly higher than those of the good sleepers (*t*_386_ = −14.34, *p*<0.01), but were not significantly different from those of the insomnia patients (*t*_1100_ = −1.88, *n.s.*). Therefore, the DBAS scores of our study sample were thought to not differ significantly from those of insomnia patients in general.

Previous studies have demonstrated a relationship between sleep-related dysfunctional beliefs and sleep variables (e.g., sleep quality and sleep efficiency) and that reductions in maladaptive beliefs lead to improved sleep [13], [14]. Similarly, the present study revealed a significant correlation between DBAS and AIS scores, but the association between these two variables was observed only at baseline. These results are somewhat in line with those of a previous cross-sectional study [26]. DBAS scores also decreased after CBT-I, but analyses such as these inevitably lead to the question of whether changes in sleep-related dysfunctional beliefs play a pivotal role in improving insomnia with CBT-I.

Schwartz and Carney [15] have alluded to the possibility that mediational analyses could yield further directions in ascertaining whether improvement of insomnia is in fact a result of changes in sleep-related beliefs. The present study was the first of its kind to analytically examine the cause-and-effect relationship between symptoms of insomnia and dysfunctional beliefs about sleep. The results indicated that change in sleep-related dysfunctional beliefs did not seem to mediate improvement in insomnia. Thus, reductions in these dysfunctional beliefs likely do not contribute significantly to improving insomnia. Given this, we speculated that changes we observed in dysfunctional beliefs about sleep might not necessarily have been the result of CBT-I, and that improvements in insomnia could have been mediated by other factors, such as change in behavior or hyperarousal states [15].

Our study had several limitations. First, the present study only examined the association between change in DBAS scores and improvement in AIS scores. As mentioned earlier, several candidate mediators of the persistence of insomnia exist other than dysfunctional beliefs, and significant interactions have been found between symptoms of insomnia and behavioral, cognitive, and hyperarousal variables [15]. Individuals receiving CBT-I are known to experience greater improvements in these aspects/variables relative to comparison groups [31], [32]. Future mediational analyses should be conducted on these variables to clarify this important issue. Second, the number of participants in this study was relatively small. Further research should be conducted with a larger sample. Third, although participants underwent PSG and/or provided self-checked sleep logs when sleep disorders other than insomnia were suspected, it is possible that some patients with a sleep-related breathing disorder or periodic limb movement disorder may have been overlooked and included in the study sample.

Finally, all participants were drawn from a single sleep disorder clinic. Therefore, the participants of our study might not be representative of the general chronic insomniac population, although the demographic data and effect size of insomnia severity were quite similar to those of previous studies [13], [33], [34].

In summary, the sleep-related dysfunctional beliefs endorsed by patients with chronic insomnia were decreased through CBT-I, but changes in these beliefs did not play a crucial role in reducing the severity of insomnia. Therefore, the impact of cognitive-behavioral approaches such as the CBT-I on behavioral and hyperarousal aspects should be studied in greater detail in order to clarify underlying mechanisms by which insomnia is significantly improved. It would also be important to examine potential long-term mediating effects of dysfunctional beliefs and attitudes about sleep on AIS scores and whether CBT-I outcomes in individual therapy setting differ from those of group therapy settings in future research.
